# The influence of non-clinical pharmacists’ understanding of and attitudes towards pharmaceutical care on their willingness to serve as clinical pharmacists in China

**DOI:** 10.1186/s12913-022-07734-8

**Published:** 2022-04-12

**Authors:** Chuchuan Wan, Yuankai Huang, Lei Chen, Xiaoyu Xi

**Affiliations:** The Research Center of National Drug Policy & Ecosystem, China Pharmaceutical University, No. 639 longmian Avenue, Jiangning District, Nanjing City, 211198 Jiangsu Province China

**Keywords:** Attitudes towards pharmaceutical care, Ordered logistic regression, Pharmacists, Role Expansion, Willingness to serve as clinical pharmacists

## Abstract

**Background:**

The shortage of clinical pharmacists is severe in China, and transferring non-clinical pharmacists into clinical pharmacists serves as a feasible solution to this problem. In China, a one-year training programme is available for non-clinical pharmacists, and those who have finished the programme are certificated as clinical pharmacists. However, not all non-clinical pharmacists are willing to serve as clinical pharmacists, and their willingness to serve as clinical pharmacists may be related to their attitudes towards pharmaceutical care. This study aims to test whether non-clinical pharmacists’ attitudes towards pharmaceutical care is positively correlated with their willingness to serve as clinical pharmacists.

**Methods:**

A cross-sectional survey was conducted in secondary and tertiary hospitals in China to collect non-clinical pharmacists’ basic demographic information, attitudes towards pharmaceutical care and willingness to serve as clinical pharmacists. An ordered logistic regression analysis was performed to test the relationship between non-clinical pharmacists’ attitudes towards pharmaceutical care and their willingness to serve as clinical pharmacists.

**Results:**

One thousand five hundred eighty non-clinical pharmacists from 755 hospitals were invited to participate in the study. Finally, 1308 valid responses were obtained and the response rate reached 82.78%. The regression results (*R*^2^ = 0.052, chi-square = 174.024, *p* < 0.0001) suggested that attitudes towards pharmaceutical care had a positive impact on non-clinical pharmacists’ willingness to serve as clinical pharmacists (*p* < 0.01). Control variables, such as age (*p* < 0.05), marital status (*p* < 0.05), professional title (*p* < 0.1), educational level (*p* < 0.1), salary expectations (*p* < 0.01) and experience providing pharmaceutical care (*p* < 0.01), also influenced non-clinical pharmacists’ willingness to serve as clinical pharmacists.

**Conclusions:**

Based on the results, suggestions are made to increase non-clinical pharmacists’ willingness to serve as clinical pharmacists.

**Supplementary Information:**

The online version contains supplementary material available at 10.1186/s12913-022-07734-8.

## Background

Pharmaceutical care (PC) is the pharmacist’s contribution to the care of individuals in order to optimize medicines use and improve health outcomes [1]. In the United States of America, Canada and other countries, the role transition of pharmacists is quite successful. Most pharmacists there not only provide drugs, but also collaborate with doctors in the process of providing PC [2]. Comparatively, China is still at the early stage of PC provision. The philosophy of PC signifies the need to transfer pharmacy practice from ensuring drug supply to providing patient-centred care [3]. As the main task of pharmacy change, pharmacists are encouraged to take on more clinical roles, such as practice pharmaceutical consultation, medication compliance guarantee, medication efficacy monitoring and medication information record feedback, while many pharmacists still regard drug supply as their main task and clinical pharmacists is insufficiently trained. The *Provisions on the Administration of Pharmaceutical Affairs in Medical Institutions* require that secondary and tertiary hospitals must be equipped with 3 and 5 clinical pharmacists, respectively. However, many hospitals fail to meet the requirement, thus there is a severe shortage of clinical pharmacists in China. In response to current situation, a one-year training programme is provided for non-clinical pharmacists, and those who complete the programme are certificated as clinical pharmacists [4, 5]. Compared with other ways, such as full-time higher education, the transition of non-clinical pharmacists to clinical pharmacists can not only relieve the pressure of the shortage of clinical pharmacists in a short time, but also rationalize the personnel of medical institutions. Therefore, selecting and training pharmacists who are willing to transfer to clinical pharmacists is very important.

There are many factors that affect the transition of non-clinical pharmacists to clinical pharmacists. Understanding of and attitudes toward PC were widely considered as two of them. Pharmacists’ understanding of PC is their perspectives of the definition, primary goals, value, contents, provision pattern and required resources of PC, and their attitudes toward PC is the extent that they accept the desired and eventual outcomes of PC, as well as its processes [6]. Improving pharmacists’ understanding of PC can clarify their responsibilities and roles. Improving pharmacists’ attitudes toward PC can enhance their professional capabilities. Thus, studying these two factors is beneficial to selecting and training pharmacists, optimizing the composition of medical institutions, and alleviating the shortage of clinical pharmacists. However, research on understanding of and attitudes towards PC is scarce in the world, even no research has clearly shown that Chinese pharmacists’ understanding of and attitudes toward PC have influence on their willingness to transfer to clinical pharmacists. El Hajj et al [7]. Conducted a cross sectional survey to investigated Qatar pharmacists’ understanding, attitudes, practice and perceived barriers related to providing PC. Ngorsuraches et al [8]. Studies Thai pharmacists’ understanding of and attitudes towards PC Xi et al [9]. Studied Chinese community pharmacists’ understanding of and attitudes towards PC.

In order to alleviate the shortage of clinical pharmacists and promote the development of pharmacists, the study aims to investigate Chinese pharmacists’ understanding of and attitudes toward PC and study the impact of the two factors on pharmacists’ willingness to serve as clinical pharmacists.

## Methods

### Study design and participants

In China, the health care system can be roughly divided into three levels: primary health care institutions, secondary hospitals and tertiary hospitals [10]. Primary health care institutions mainly provide health care for patients with relatively stable health status and do not need to consume too much medical resources. Secondary hospitals mainly provide health care for patients with improvement referred from tertiary hospitals or patients with aggravation referred from primary health care institutions. Tertiary hospitals mainly provide health care for acute or critical patients nationwide, accept patients with aggravation referred from secondary hospitals, provide technical guidance to subordinate hospitals, train medical professionals and carry out scientific research projects. Therefore, clinical pharmacy services, mainly provided by tertiary hospitals [10], are poorly developed in secondary hospitals, not to mention in primary health care institutions [1, 11, 12]. So, currently pharmacists and the demand for clinical pharmacists mainly come from secondary and tertiary hospitals in China. Therefore, the study only investigated non-clinical pharmacists in secondary and tertiary hospitals.

The inclusion criteria of the study were as follows: (1) full-time non-clinical pharmacist of the hospitals; (2) able to participate in the study and willing to sign the informed consent document. The exclusion criteria were as follows: part-time pharmacists and intern pharmacists were excluded from the sample.

A stratified sampling strategy was adopted, and the detailed strategies were as follows. The sample of the study came from 31 provinces administrative regions in China. Firstly, the cities or districts of each regions were classified into three groups, according to their gross domestic product per capita in 2018 [13, 14], forming a total of 93 groups. Secondly, at least two local secondary hospitals and two local tertiary hospitals were selected for convenience sampling in each group. Thirdly, at least two full-time pharmacists were selected for convenience sampling from each hospital.

### Variables and instruments

The questionnaire which consists of three sections was designed with reference to the opinions of 7 experts from hospitals and universities, written in Chinese.

In the first section, objective demographic information was collected including gender, age, marital status, number of children, working years, educational level, professional background, professional title. These factors may affect the willingness of pharmacists to transfer to clinical pharmacists., so they were included in the study as control variables.

In the second section, some subjective information about the potential factors which influence the non-clinical pharmacists’ willingness to serve as clinical pharmacists was collected, including their understanding of and attitudes towards PC, salary expectations (whether they thought clinical pharmacists had a higher salary than non-clinical pharmacists) and previous experiences of providing PC. Pharmacists’ attitudes towards PC were measured by the PC Attitude Scale (PCAS), which is a 13-item scale with a five level Likert scale [9, 15, 16]. The attitudes of pharmacists towards PC were included in the study as an independent variable and the other information was include in the study as control variables. A description of the independent variables presumed to affect non-clinical pharmacists’ willingness to serve as clinical pharmacists is provided in Table 1.

**Table 1:** Independent variables hypothesized as affecting non-clinical Pharmacists’ Willingness

Variables	Coding
Gender^a^	0 = male, 1 = female
Age^b^	Pharmacist’s age
Marital status^a^	0 = unmarried, 1 = married, 2 = other status (divorced, widowed or other status)
Number of children^b^	Number of children the pharmacist has
Working years^b^	Length (years) the pharmacist has worked in this industry
Educational level^a^	0 = below bachelor’s degree, 1 = bachelor's degree, 2 = master's degree, 3 = doctor's degree
Professional background^a^	0 = nursing-related, 1 = medication-related, 2 = pharmacy-related, 3 = clinical pharmacy
Professional title^a^	0 = junior level, 1 = intermediate level, 2 = vice-senior level, 3 = senior level
Attitudes towards PC^b^	The score of each item was summed (the highest possible score was 65, and the lowest possible score was 13)
Do you think the clinical pharmacist salary is higher than yours? ^a^	0 = no, I don’t; 1 = I don’t know; 2 = yes, I do
Do you have experience providing PC to patients? ^a^	0 = never, 1 = seldom, 2 = not sure, 3 = sometimes, 4 = often

^a^nominal variable

^b^linear variable

In the third section, the respondents’ willingness to serve as clinical pharmacists was collected. The dependent variable, i.e., non-clinical pharmacists’ willingness to serve as clinical pharmacists, was measured by the question, “To what extent are you willing to join the clinical pharmacist training programme and serve as a clinical pharmacist?” The response options were “unwilling”, “somewhat unwilling”, “not sure”, “somewhat willing” and “very willing”.

The validity, rationality, comprehensibility and readability of the questionnaire had been verified by experts and the results of a pilot survey in three tertiary hospitals in Nanjing, Jiangsu province, China. Based on the feedback of the pilot survey, the research team revised the questionnaire and formulate the final version.

### Data collection

The survey was conducted from July to August 2019. A total of 500 undergraduate students majoring in clinical pharmacy or pharmacy were recruited as data collectors and at least four data collectors were assigned to each group. All data collectors were trained so that they could understand the background, purposes and methods of the study.

When they arrived at the chosen hospital, data collectors first approached the administrators of the hospital and asked for permission to conduct the survey. After permission was obtained, the data collectors went to identify potential participants and told them about the purpose and content of the survey. Potential participants signed an informed consent form if they agreed to participate in the survey. Then, data collectors provided the participants with an electronic device on which Interview Master, a survey app, had been installed and gave them instructions about how to complete the questionnaire on the app. The participants were asked to complete the questionnaire alone and the data collectors were not allowed to provide any view on the questionnaire, but only the requirements or instructions of questionnaire filling. After completing the questionnaire, the data would be fed back to the auditors through the app immediately. If auditors found that there were obvious errors in the data, the data would be returned to the data collectors, who would verify it with the respondents. A total of 5 master students were recruited and trained as auditors.

### Data analysis

The online electronic data were imported into SPSS 24.0 and checked by two researchers sequentially for data verification. Incomplete and obviously mistaken data were replaced with the mean value or excluded if they could not be replaced.

Ordered regression analysis was performed, as the willingness to serve as a clinical pharmacist was rated, and many factors could influence non-clinical pharmacists’ willingness to serve as clinical pharmacists. We tested the multicollinearity through the "coldiag2" command in Stata. The significance level was defined as *p* < 0.01. Data analysis was performed with Stata 15.0.

## Results

One thousand five hundred eighty non-clinical pharmacists from 755 hospitals were invited to participate in the study. Excluding the participants who withdrew halfway, a total of 1353 non-clinical pharmacists participated in the survey. After data screening and cleaning, 1308 valid cases were collected. The response rate reached 82.78%. The average age of the participants was 34.5 years, the average working years of the participants was 10.64, the ratio of male to female pharmacists was approximately 0.47 and most of the participants were married (75.00%) and have one child (54.90%). Most of the participants had a bachelor's degree (69.80%) and only 0.3% have a doctoral degree. In this study, respondents with junior titles accounted for 55.00% of the participants. Most of the respondents graduated from pharmacy-related majors (82.50%), and there were a few people with medical (3.80%) and clinical pharmacy backgrounds (3.50%) (see Table 2).

**Table 2:** Demographics of the Participants (*n* = 1308)

Characteristics	Frequency	Percentage
**Gender**		
Male	419	32.00%
Female	889	68.00%
**Age**	34.53 (Mean)	7.79 (S.D.)
**Marital status**		
Unmarried	307	23.70%
Married	982	74.80%
Other status (divorced or widowed)	19	1.50%
**Number of children**		
No children	407	31.10%
One child	718	54.90%
Two children	177	13.50%
Three children	6	0.50%
**Education**		
Below bachelor’s degree	244	18.10%
Bachelor’s degree	913	69.80%
Master’s degree	147	11.20%
Doctoral degree	4	0.30%
**Working years**	10.64 (Mean)	8.38 (S.D.)
**Professional title**		
Junior	720	55.00%
Intermediate	474	36.20%
Vice-senior	103	7.90%
Senior	11	0.80%
**Professional background**		
Clinical pharmacy	48	3.50%
Pharmacy-related	1116	82.50%
Medication-relation	52	3.80%
Nursing-related	26	1.90%

The survey results show that overall, pharmacists’ understanding of PC was at an intermediate level (see Table 3). Although most pharmacists had clear understanding of the main purpose and significance of PC, and the resource support required by pharmacy services, their understanding of the responsible subject of pharmacy services was not clear. The proportion of correct answer to the expression " PC providers are directly responsible for the patient’s health outcomes " was only 45.00%, indicating that more than half of the participants did not have a clear understanding of the responsible subject of PC and believed that clinical pharmacists were not responsible for the treatment results of patients. Regarding the negative expression of "pharmaceutical service is a medication consultation service", the correct answer rate was only 49.70%, indicating that the respondents were not clear about the specific content of PC. The vast majority of respondents did not know "whether a consultation room or private area is needed to provide PC, and the correct rate was only 27.10%, which explains to a certain extent indicates that there is a lack of clear understanding of PC in the aspects of major content, specific forms of providing PC.

**Table 3:** Pharmacists’ understanding of PC

Statement	n (%) Correct answer
1) PC providers are directly responsible for the patient’s health outcomes	588(45.00)
2) The primary aim of PC is to improve and maintain the patient’s quality of life	1138(87.00)
3) PC is just a medication counseling service[R]	650(49.70)
4) The term clinical pharmacy is interchangeable with PC[R]	1096 (83.80)
5) PC is an extension of the current pharmacy services	1083 (82.80)
6) In PC the pharmacist identifies and manages a patient’s existing and other potential drug therapy problems	1220(93.30)
7) PC involves a defined process of activities, all steps of which must be completed in order to provide this service	960(74.30)
8) All patients prescribed medicines require PC services	876(67.00)
9) PC requires availability of drug information resources	1284(98.20)
10) To provide PC a consultation room or private area must be available[R]	354(27.10)
11) Provision of PC offers a feedback mechanism that optimizes the use of medicinal products	1270(97.10)
12) The patient’s active involvement is optional in the provision of PC[R]	1122(85.80)

R item with a reverse statement

Participants generally had positive attitudes toward PC. Specifically in terms of attitudes toward the value and benefits of PC, the vast majority of respondents have a positive attitude towards the value of PC. 95.90% agreed with the statement of item 5 " I think the practice of PC is valuable ". 94.80% agreed that " Providing PC is professionally rewarding ", 92.70% agreed that " I feel that PC is the right direction for the profession to be headed toward", 86.50% of the respondents agreed that " I feel that the PC movement will benefit pharmacists ", 91.90% of the respondents agreed that " I feel that the PC movement will improve patient health", and even nearly 96.00% of the respondents agreed that “I feel that practicing PC would benefit my professional career as a pharmacy practitioner”. It can be seen that the vast majority of respondents have a positive attitude towards the value of PC and recognize the value of PC in promoting the personal professional development of hospital pharmacy workers, promoting the development of hospital pharmacy, and improving the result of patient care (see Table 4).

**Table 4:** Participants’ Scores on the PC Attitude Scale (*n* = 1308)

Item	Agree and strongly agree (%)	Mean	S.D
1) All pharmacists should perform PC	70.10	3.74	0.945
2) The primary responsibility of pharmacists in healthcare settings should be to prevent and solve medication-related problems	73.70	3.74	0.830
3) Pharmacists’ primary responsibility should be to practice PC	67.00	3.61	0.893
4) Pharmacy students can perform PC during their clerkship	59.10	3.44	0.979
5) I think the practice of PC is valuable	95.90	4.31	0.618
6) Providing PC takes too much time and effort [R]	52.50	3.30	1.007
7) Providing PC is not worth the additional workload that it places on the pharmacist [R]	15.60	2.34	0.994
8) I would like to perform PC as a pharmacist practitioner	91.20	4.12	0.624
9) Providing PC is professionally rewarding	94.80	4.21	0.587
10) I feel that PC is the right direction for the profession to be headed toward	92.70	4.19	0.602
11) I feel that the PC movement will benefit pharmacists	86.50	4.13	0.625
12) I feel that the PC movement will improve patient health	91.90	4.16	0.610
13) I feel that practicing PC would benefit my professional career as a pharmacy practitioner	95.90	4.21	0.546

R item with a reverse statement

While pharmacists generally recognize the value of PC, they also realize that providing PC requires additional time and energy. More than half of the respondents (52.50%) think that providing pharmaceutical services requires too much time and energy, but only 15.60% think that it is not worthwhile for hospital pharmacists to increase their workload to carry out PC. This shows that non-clinical pharmacists are aware the additional time and energy required to provide pharmacy care, but also recognizes that the time and energy is worthwhile (see Table 4).

After weighing the value of PC and the additional time and energy required, hospital pharmacists’ attitudes towards the responsibilities of clinical pharmacists and the practice pf PC are not clear. Different opinions on the responsible subject of PC were collected. About 1/3 of pharmacists believed that carrying out pharmaceutical services is only the responsibility of clinical pharmacists, 33.00% of pharmacists do not think that the main responsibility of hospital pharmacists is to carry out PC, and 29.90% of pharmacists do not think that providing PC is the responsibility of all hospital pharmacists. 59.10% of the respondents believe that clinical pharmacy students can provide pharmacy services during internships. The above view obviously does not coincide with the general trend of hospital pharmacy which is directing toward the provision of patient-centered care. There are still certain practical obstacles to the implementation of the duties of clinical pharmacists in China (see Table 4).

The ordered logistic regression results showed that *R*^2^ = 0.052, and the chi-square was 174.024, *p* < 0.0001, indicating the good fit of the model. The multicollinearity test results showed that the condition number is 50.89(> 30 but < 100), so there was a certain degree of multicollinearity between the independent variables. But it had not affected the regression and interpretation of the model. The main variables that produce the multicollinearity problem were age, working years, marital status and experience of providing PC. The results supported our presumption that non-clinical pharmacists’ willingness to serve as clinical pharmacists was positively related to their attitudes towards PC. In addition, control variables, such as age, marital status, professional title, educational level, professional background, salary expectations and experience providing PC, also influenced non-clinical pharmacists’ willingness to serve as clinical pharmacists (see Table 5).

**Table 5:** Results of the ordered logistic regression

Willingness	OR	St.Err	z	*P*-value	[95% Conf	Interval]
**Gender**						
Male (control group)						
Female	1.062	0.119	0.537	0.591	0.853	1.322
**Age**	0.975	0.013	-1.978	0.048**	0.950	1.000
**Marital status**						
Unmarried (control group)						
Married	0.760	0.104	-2.004	0.045**	0.580	0.994
Divorced or widowed	0.723	0.315	-0.745	0.456	0.307	1.698
**Number of children**	1.124	0.105	1.255	0.209	0.936	1.350
**Working years**	1.010	0.012	0.864	0.388	0.987	1.033
**Professional title**						
Junior (control group)						
Intermediate	0.857	0.103	-1.289	0.198	0.678	1.084
Vice-senior	0.673	0.141	-1.894	0.058*	0.447	1.014
Senior	1.547	0.963	0.702	0.483	0.457	5.239
**Do you think the clinical pharmacist salary is higher than yours’?**						
No, I don’t (control group)						
I don’t know	0.816	0.124	-1.345	0.179	0.606	1.098
Yes, I do	1.492	0.220	2.709	0.007***	1.117	1.993
**Do you have experience providing PC to patients?**						
Never (control group)						
Seldom	2.275	0.671	2.788	0.005***	1.277	4.056
Not sure	1.997	0.707	1.954	0.051*	0.998	3.996
Sometimes	2.622	0.740	3.418	0.001***	1.509	4.557
Often	3.939	1.118	4.829	0.000***	2.258	6.871
**Educational level**						
Below bachelor’s degree (control group)						
Bachelor's degree	0.786	0.110	-1.720	0.085*	0.598	1.034
Master's degree	0.677	0.137	-1.932	0.053*	0.455	1.006
Doctor's degree	0.409	0.368	-0.995	0.320	0.070	2.382
**Professional background**						
Nursing-related (control group)						
Medication-related	2.011	0.874	1.608	0.108	0.858	4.714
Pharmacy-related	1.448	0.509	1.054	0.292	0.728	2.883
Clinical pharmacy	1.128	0.487	0.279	0.781	0.484	2.631
Others	2.814	1.645	1.769	0.077*	0.895	8.851
**Attitudes towards PC**	1.102	0.013	8.025	0.000***	1.076	1.129
Cut	0.830	0.824			-0.785	2.444
Cut	2.408	0.810			0.821	3.996
Cut	3.640	0.811			2.051	5.230
Cut	5.423	0.819			3.818	7.028
Mean dependent var	3.028	SD dependent var				1.006
Pseudo r-squared	0.052	Number of obs				1308.000
Chi-square	174.024	Prob > chi2				0.000
Akaike crit. (AIC)	3256.457	Bayesian crit. (BIC)				3396.216

^***^ p < 0.01, ** p < 0.05, * p < *0.1*

A robustness check was conducted by replacing the dependent variable “number of children” with four different variables that specifically measured the number of children of different ages: number of children (0 ~ 6 years old), number of children (7 ~ 12 years old), number of children (13 ~ 17 years old), and number of children (older than 18). The results remained robust after this check (the result of the robustness check is available in the Appendix).

## Discussion

The study aims to determine the relationship between non-clinical pharmacists’ attitudes towards PC and their willingness to serve as clinical pharmacists. This is the first nationwide study on this subject in China. The sample covers all provinces of mainland China, and the sample size is sufficient. So the representativeness of the sample is acceptable. The regression results show that non-clinical pharmacists’ attitudes towards PC had a positive impact on non-clinical pharmacists’ willingness to serve as clinical pharmacists. Control variables, including age, marital status, professional title, educational level, professional background, salary expectations, and experience providing PC, also influenced non-clinical pharmacists’ willingness to serve as clinical pharmacists.

First, our hypothesis that non-clinical pharmacists’ attitudes towards PC are positively correlated with their willingness to serve as clinical pharmacists is supported by the regression results. This provides important insight for solutions to the shortage of clinical pharmacists. In China, the role of clinical pharmacists and the importance of PC are often underestimated [17]. Measures should be taken to improve people’s understanding of and attitudes towards PC, as well as people’s perceptions of the role of clinical pharmacists [9]. For example, more empirical studies should be conducted to recognize the value of PC and the role of clinical pharmacists; laws and regulations should be implemented to explicitly define the role of clinical pharmacists; and hospital administrators should realize the importance of PC and recognize the role of clinical pharmacists in the provision of PC.

Second, younger non-clinical pharmacists were more interested in joining clinical pharmacist training programs and serving as clinical pharmacists, which supports the findings of Schafheutle et al. [18]. Although young staff are more open-minded and willing to try new things, they are often less experienced than older staff. This means that efforts should be made not only to increase younger staff members’ willingness to serve as clinical pharmacists but also to attract more older staff members with rich working experience.

Third, marital status had an impact on non-clinical pharmacists’ willingness to serve as clinical pharmacists. Married non-clinical pharmacists were less willing than unmarried non-clinical pharmacists to become clinical pharmacists. This could be explained by the fact that 68% of the respondents were female, and females often are more devoted to their families and encounter greater conflicts between family and work [19]. This indicates the importance of ensuring clinical pharmacists’ job security. Measures such as providing a family-supportive environment [19] and building a career ladder [20] to guide career promotion should be taken to increase the job security of clinical pharmacists.

Fourth, non-clinical pharmacists who thought that clinical pharmacists have a higher salary than non-clinical pharmacists were more willing to serve as clinical pharmacists, which supports many previous findings [21–23] This implies that salary is an important factor influencing non-clinical pharmacists’ career choices. Considering that clinical pharmacists are responsible for PC services without financial support in China [24], local authorities and hospital administrators should make efforts to ensure clinical pharmacists are paid properly. For example, measures such as provide subsidies for clinical pharmacists who provide PC, charge fees for PC services could be taken.

In addition, educational level influenced non-clinical pharmacists’ willingness to serve as clinical pharmacists. Non-clinical pharmacists with a bachelor’s degree or master’s degree were more willing than non-clinical pharmacists with educational levels lower than a bachelor’s to serve as clinical pharmacists. The minimum requirement for clinical pharmacists (a bachelor’s degree) serves as a threshold, and those who do not meet this requirement can participate in continuing education to improve themselves [25].

This study also showed that non-clinical pharmacists with a vice-senior professional title were less willing than junior non-clinical pharmacists to serve as clinical pharmacists, likely because vice-senior professionals had many achievements in their current positions, and it was risky for them to change jobs. However, as vice-senior professionals are often more experienced than junior non-clinical pharmacists, the inclusion of vice-senior professionals on clinical pharmacist teams would contribute greatly to team construction. Therefore, it is necessary to strengthen occupational convergence and development for non-clinical pharmacists with higher professional levels. Interestingly, there was no difference between those with senior professional titles and the control group. We expect that this is because only 11 of the 1308 participants had senior professional titles, accounting for only 0.8% of the sample.

Regarding the influence of professional background, interestingly, non-clinical pharmacists with other professional backgrounds were more willing than those in the nursing-related group to serve as clinical pharmacists, which we cannot adequately explain. We assume this was caused by unobserved bias, which requires further evidence.

The limitations of the study are as follows. First, the control variables were chosen based on the results of a literature review. Other unobserved factors may influence non-clinical pharmacists’ willingness to serve as clinical pharmacists. Future studies may include other control variables. Meanwhile in regression analysis, there is a certain degree of collinearity among the independent variables, which may affect the results. Second, this study focused on non-clinical pharmacists’ willingness to serve as clinical pharmacists, but their willingness does not mean they would actually become clinical pharmacists. Transforming willingness into action was too complicated a question to discuss in this study. Third, there are information biases in study. To limit information biases, the following efforts had been made: At the stage of questionnaire designing, objective indicators were included in the questionnaire as much as possible. Then, we had designed a strictly standardized research process, including the sequence and content of each link. Finally, 5 master students were recruited and trained as auditors to review data and guarantee data quality in time. However, these measures can not completely avoid information biases.

## Conclusions

A cross-sectional survey was conducted, and a logistic regression analysis was performed to test the relationship between non-clinical pharmacists’ attitudes and their willingness to serve as clinical pharmacists. It was found that attitudes towards PC had a positive impact on willingness to serve as clinical pharmacists. Control variables, such as age, marital status, professional title, educational level, professional background, salary expectations, and experience providing PC, also influenced non-clinical pharmacists’ willingness to serve as clinical pharmacists. Based on these results, we suggest emphasising the role of clinical pharmacists, attracting more experienced non-clinical pharmacists, and increasing job security to increase non-clinical pharmacists’ willingness to serve as clinical pharmacists.

## Supplementary Information

**Additional file 1. **

## Data Availability

The datasets generated and/or analysed during the current study are not publicly available, because data used in this research is part of a larger data set exclusively constructed with the authors’ efforts, and this data set is to be used in the authors’ other researches, which required confidentiality. but they are available from the corresponding author on reasonable request.

## Abbreviation

PC: Pharmaceutical care
