# Supplementary material for: The influence of non-clinical pharmacists’ understanding of and attitudes towards pharmaceutical care on their willingness to serve as clinical pharmacists in China
Source: BMC Health Serv Res. 2022 Apr 12;22:484. doi: 10.1186/s12913-022-07734-8 (PMC9004027; doi:10.1186/s12913-022-07734-8)
Supplement: Supplementary file 1 — Additional file 1. [file 12913_2022_7734_MOESM1_ESM.docx]

Appendix: Robustness Check Through Replacement of the Control Variables

| Willingness | | | OR | St.Err | z | p-value | | [95% Conf | | Interval] |
| --- | --- | --- | --- | --- | --- | --- | --- | --- | --- | --- |
| **Gender** | | |  |  |  |  | |  | |  |
| Male (control group) | | |  |  |  |  | |  | |  |
| Female | | | 1.067 | 0.119 | 0.583 | 0.560 | | 0.857 | | 1.329 |
| **Age** | | | 0.971 | 0.014 | -1.986 | 0.047^**^ | | 0.943 | | 1.000 |
| **Marital status** | | |  |  |  |  | |  | |  |
| Unmarried (control group) | | |  |  |  |  | |  | |  |
| Married | | | 0.767 | 0.105 | -1.926 | 0.054^*^ | | 0.586 | | 1.005 |
| Divorced or widowed | | | 0.695 | 0.303 | -0.834 | 0.404 | | 0.295 | | 1.635 |
| **Number of children** | | |  |  |  |  | |  | |  |
| Number of children (0~6 years old) (control group) | | | 1.161 | 0.115 | 1.504 | 0.133 | | 0.956 | | 1.409 |
| Number of children (7~12 years old) | | | 1.054 | 0.166 | 0.333 | 0.739 | | 0.774 | | 1.436 |
| Number of children (13~17 years old) | | | 1.076 | 0.216 | 0.367 | 0.714 | | 0.727 | | 1.594 |
| Number of children (>18 years old) | | | 1.399 | 0.310 | 1.518 | 0.129 | | 0.907 | | 2.159 |
| **Working years** | | | 1.008 | 0.012 | 0.677 | 0.498 | | 0.985 | | 1.031 |
| **Professional title** | | |  |  |  |  | |  | |  |
| Junior (control group) | | |  |  |  |  | |  | |  |
| Intermediate | | | 0.861 | 0.103 | -1.252 | 0.210 | | 0.680 | | 1.089 |
| Vice-senior | | | 0.674 | 0.141 | -1.889 | 0.059^*^ | | 0.447 | | 1.015 |
| Senior | | | 1.566 | 0.972 | 0.723 | 0.470 | | 0.464 | | 5.285 |
| **Do you think the clinical pharmacist salary is higher than yours’?** | | |  |  |  |  | |  | |  |
| No, I don’t (control group) | | |  |  |  |  | |  | |  |
| I don’t know | | | 0.827 | 0.126 | -1.251 | 0.211 | | 0.614 | | 1.114 |
| Yes, I do | | | 1.501 | 0.222 | 2.743 | 0.006^***^ | | 1.123 | | 2.005 |
| **Do you have experience providing PC to patients?** | | |  |  |  |  | |  | |  |
| Never (control group) | | |  |  |  |  | |  | |  |
| Seldom | | | 2.340 | 0.691 | 2.879 | 0.004^***^ | | 1.312 | | 4.175 |
| Not sure | | | 2.014 | 0.712 | 1.979 | 0.048^**^ | | 1.007 | | 4.027 |
| Sometimes | | | 2.657 | 0.749 | 3.466 | 0.001^***^ | | 1.529 | | 4.617 |
| Often | | | 4.010 | 1.138 | 4.893 | 0.000^***^ | | 2.299 | | 6.996 |
| **Educational level** | | |  |  |  |  | |  | |  |
| Below bachelor’s degree (control group) | | |  |  |  |  | |  | |  |
| Bachelor's degree | | | 0.799 | 0.112 | -1.595 | 0.111 | | 0.606 | | 1.053 |
| Master's degree | | | 0.688 | 0.140 | -1.840 | 0.066^*^ | | 0.462 | | 1.025 |
| Doctor's degree | | | 0.357 | 0.321 | -1.144 | 0.253 | | 0.061 | | 2.086 |
| **Professional background** | | |  |  |  |  | |  | |  |
| Nursing-related (control group) | | |  |  |  |  | |  | |  |
| Medication-related | | | 1.980 | 0.865 | 1.564 | 0.118 | | 0.841 | | 4.660 |
| Pharmacy-related | | | 1.429 | 0.503 | 1.015 | 0.310 | | 0.717 | | 2.848 |
| Clinical pharmacy | | | 1.103 | 0.478 | 0.227 | 0.821 | | 0.472 | | 2.578 |
| Others | | | 2.666 | 1.569 | 1.666 | 0.096^*^ | | 0.841 | | 8.451 |
| **Attitudes towards PC** | | | 1.103 | 0.013 | 8.055 | 0.000^***^ | | 1.077 | | 1.129 |
| Cut | | | 0.763 | 0.843 |  |  | | -0.890 | | 2.415 |
| Cut | | | 2.343 | 0.829 |  |  | | 0.718 | | 3.968 |
| Cut | | | 3.578 | 0.830 |  |  | | 1.950 | | 5.205 |
| Cut | | | 5.362 | 0.838 |  |  | | 3.720 | | 7.004 |
|  |  | |  | |  |  |  | |  | |
| Mean dependent var | | 3.028 | | SD dependent var | | |  | | 1.006 | |
| Pseudo r-squared | | 0.052 | | Number of obs | | |  | | 1308.000 | |
| Chi-square | | 176.436 | | Prob > chi2 | | |  | | 0.000 | |
| Akaike crit. (AIC) | | 3260.045 | | Bayesian crit. (BIC) | | |  | | 3415.333 | |

**** p<0.01, ** p<0.05, * p<0.1*
